# How does Poisson kriging compare to the popular BYM model for mapping disease risks?

**DOI:** 10.1186/1476-072X-7-6

**Published:** 2008-02-04

**Authors:** Pierre Goovaerts, Samson Gebreab

**Affiliations:** 1 BioMedware, Inc., Ann Arbor, MI, USA; 2 Utah State University, Department of Watershed Sciences, Logan, UT, USA

## Abstract

**Background:**

Geostatistical techniques are now available to account for spatially varying population sizes and spatial patterns in the mapping of disease rates. At first glance, Poisson kriging represents an attractive alternative to increasingly popular Bayesian spatial models in that: 1) it is easier to implement and less CPU intensive, and 2) it accounts for the size and shape of geographical units, avoiding the limitations of conditional auto-regressive (CAR) models commonly used in Bayesian algorithms while allowing for the creation of isopleth risk maps. Both approaches, however, have never been compared in simulation studies, and there is a need to better understand their merits in terms of accuracy and precision of disease risk estimates.

**Results:**

Besag, York and Mollie's (BYM) model and Poisson kriging (point and area-to-area implementations) were applied to age-adjusted lung and cervix cancer mortality rates recorded for white females in two contrasted county geographies: 1) state of Indiana that consists of 92 counties of fairly similar size and shape, and 2) four states in the Western US (Arizona, California, Nevada and Utah) forming a set of 118 counties that are vastly different geographical units. The spatial support (i.e. point versus area) has a much smaller impact on the results than the statistical methodology (i.e. geostatistical versus Bayesian models). Differences between methods are particularly pronounced in the Western US dataset: BYM model yields smoother risk surface and prediction variance that changes mainly as a function of the predicted risk, while the Poisson kriging variance increases in large sparsely populated counties. Simulation studies showed that the geostatistical approach yields smaller prediction errors, more precise and accurate probability intervals, and allows a better discrimination between counties with high and low mortality risks. The benefit of area-to-area Poisson kriging increases as the county geography becomes more heterogeneous and when data beyond the adjacent counties are used in the estimation. The trade-off cost for the easier implementation of point Poisson kriging is slightly larger kriging variances, which reduces the precision of the model of uncertainty.

**Conclusion:**

Bayesian spatial models are increasingly used by public health officials to map mortality risk from observed rates, a preliminary step towards the identification of areas of excess. More attention should however be paid to the spatial and distributional assumptions underlying the popular BYM model. Poisson kriging offers more flexibility in modeling the spatial structure of the risk and generates less smoothing, reducing the likelihood of missing areas of high risk.

## Background

Cancer mortality maps are used by public health officials to identify areas of excess and to guide surveillance and control activities. Interpretation of those maps is frequently hampered by the presence of noise caused by unreliable extreme rates computed from sparsely populated geographical units or for diseases with a low frequency of occurrence [1]. Ignoring the uncertainty attached to rate estimates can lead to misallocation of resources to investigate unreliable clusters of high risk, while areas of real concern might go undetected.

Over the years, statistical techniques of increasing complexity, usually involving Bayesian models, have been developed to improve the reliability of risk estimates by borrowing information from neighboring entities [2-6]. The implementation of these models in software such as WinBUGS [7] has fostered their adoption by both the statisticians and epidemiologists. Yet, the estimation of model parameters requires iterative procedures, such as Markov Chain Monte Carlo (MCMC) methods, that are computer intensive and require fine-tuning, which makes their application and interpretation challenging for non-statisticians [8-10]. This might explain why the majority of applications nowadays still rely on one of the early models introduced by Besag, York and Mollie in 1991 [5] which is commonly referred to as BYM model. Quoting the 2005 comparison paper by Best *et al*. [2]*"a (non exhaustive) search of the major epidemiological journals over the past decade indicates that the BYM model appears to be the only fully Bayesian spatial model to have been used in published applications of disease mapping outside of the statistical literature"*. This model is overwhelmingly used with the conditional auto-regressive (CAR) model for defining the random effect associated with spatial autocorrelation [2,9,11]. The arbitrary neighborhood relationship underlying the CAR model is computationally convenient but is not well-suited to situations where geographical entities have different sizes and shapes and are not arranged in a regular pattern [12]. The choice of a neighborhood weight structure (i.e. adjacency-based versus distance-based spatial weights) is also very subjective and seldom justified despite its substantial impact on the results [13]. Last, simulation studies have demonstrated the strong smoothing effect of Bayesian disease-mapping models, in particular the BYM model, which limits their ability to detect localized increases in risk [14].

Geostatistics provides an alternative, yet still little known, model-based approach to disease mapping [15]. The first initiative to tailor geostatistical tools to the analysis of disease rates must be credited to Christian Lajaunie [16] from the Center of geostatistics in Fontainebleau, France. Although it was introduced the same year as the BYM model, the geostatistical approach, called binomial cokriging, went largely unnoticed. The rare applications of the method are the study of the risk of childhood cancer in the West Midlands of England [17-19] and the mapping of lung cancer mortality across the US [20]. More recently, a similar approach, called Poisson kriging, was developed in the field of marine ecology and generalized to the analysis of cancer mortality rates and cholera incidence data [21-23]. Poisson kriging was combined with stochastic simulation to generate multiple realizations of the spatial distribution of cancer mortality risk, allowing the propagation of uncertainty through the detection of cancer clusters and outliers [24]. Point Poisson kriging, which assigns each measured rate to the geographic centroid of the unit over which it has been recorded, was later replaced by Area-to-Area (ATA) Poisson kriging that accounts for the geometry of administrative units and the spatial repartition of the population at risk in semivariogram estimation and kriging [25]. Like fully Bayesian models, Poisson kriging yields the full posterior distribution of the risk. This distribution is however assumed to be Gaussian and fully characterized by the kriging estimate and variance, while the BYM posterior distribution is modeled by the empirical distribution of simulated risk values. The trade-off cost for the simplicity of Poisson kriging is that, unlike the full Bayesian approach, the uncertainty attached to the parameters of the correlation function is ignored in the analysis, which should lead to smaller prediction variances in general [26].

Only a few comparison studies of geostatistical and Bayesian estimators have been published in the literature. In the earliest study [27], marginal and conditional generalized linear models (GLM) were compared to three implementations of universal kriging: no nugget effect, unweighted nugget effect, and a nugget effect that is weighted inversely proportional to the expected counts. Despite their simplicity, the linear kriging models provided surprisingly good predictions, in particular the third implementation that acknowledges the larger uncertainty (noise) of rates computed from small populations (fewer expected counts). Recent studies extended the comparison to semivariogram estimators and kriging algorithms that were derived specifically for count data using the properties of binomial or Poisson distributions. First, the benefit of binomial or Poisson kriging over a few simple smoothers (i.e. population-weighted estimators and empirical Bayes smoothers) was assessed under different scenarios for the disease frequency, the population size, and the spatial pattern of risk [20,22]. Simulation studies showed that Poisson kriging outperforms other approaches for most scenarios, with a clear benefit when the risk values are spatially correlated. In a third study [26], Poisson kriging was compared to Diggle *et al*.'s "model-based kriging" [28] for mapping the relative abundance of species in the presence of spatially heterogeneous observation efforts and sparse animal sightings. Both methods gave equivalent results for 90% of the predictions; differences were observed for higher values with a smoothing for the kriging. Methods differed much more in terms of the prediction variance: the lognormal hypothesis underlying the Bayesian model induced similarities between the maps of the prediction variance and estimate (i.e. proportionality), while the Poisson kriging variance mainly reflects the observation effort (i.e. lower variance for longer observation times). Because this study was conducted on real data, the underlying risk pattern was unknown, precluding any objective assessment of prediction performances.

This paper presents the first simulation-based evaluation of performance of Poisson kriging (both point and ATA implementations) and full Bayesian disease-mapping models. The benchmark for Bayesian methods is the BYM model since it is the most widely applied in the health literature. Both types of model are first illustrated using age-adjusted lung and cervix cancer mortality rates recorded for white females in two contrasted county geographies: 1) state of Indiana that consists of 92 counties of fairly similar size and shape, and 2) four states in the Western US (Arizona, California, Nevada and Utah) forming a set of 118 counties that are vastly different geographical units. Simulation studies are then conducted for the same two county geographies. Performance criteria include the magnitude of prediction errors of the underlying risk, the precision and accuracy of the model of uncertainty, and the ability to discriminate between background and high-risk areas.

## Methods

### Cancer data sets

The basic properties of Poisson kriging and Bayesian model will be illustrated using directly age-adjusted mortality rates for a frequent (lung) and less frequent (cervix) cancer. These data were described in a previous study [25] and only their most salient features are summarized here. The analysis focuses on white female rates recorded over the 1970–1994 period and adjusted using the 1970 population pyramid. Two areas with contrasted county geographies were considered: 1) state of Indiana (Region 1), and 2) four states in the Western US (Arizona, California, Nevada and Utah) that will be referred to as Region 2. The choice of these two specific geography areas was guided by the need to compare performances in two contrasted settings: 1) all geographical units have a fairly similar size and shape, which is the "ideal" situation for point Poisson kriging or Bayesian methods implemented under the conditional auto-regressive (CAR) model that ignores the spatial support of the data, and 2) geographical units display a wide range of sizes and shapes, which should favour Area-to-Area Poisson kriging that implicitly accounts for the spatial support of the data in the analysis. The West coast provides a perfect example for the second type of geography (i.e. set of 118 vastly different counties), while Indiana includes a reasonable number (i.e. 92) of counties that are geometrically fairly similar.

**Figure 1:**
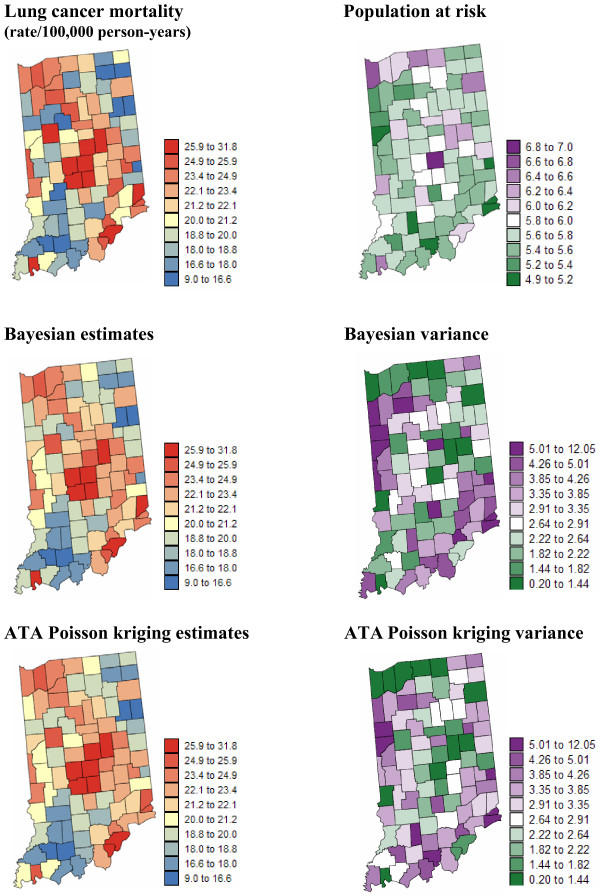
**Maps of age-adjusted lung cancer mortality rates and the risk estimated using Bayesian and geostatistical methods**. The fill color in the left column maps represents the age-adjusted mortality rate per 100,000 person-years recorded over the period 1970–1994 (top graph) or the risk estimated using a Bayesian approach (BYM model) or ATA Poisson kriging. The class boundaries correspond to the deciles of the histogram of original rates. The corresponding prediction variance is mapped in the right column, along with the population at risk that was back-calculated from the rate and count data (lognormal scale).

**Figure 2:**
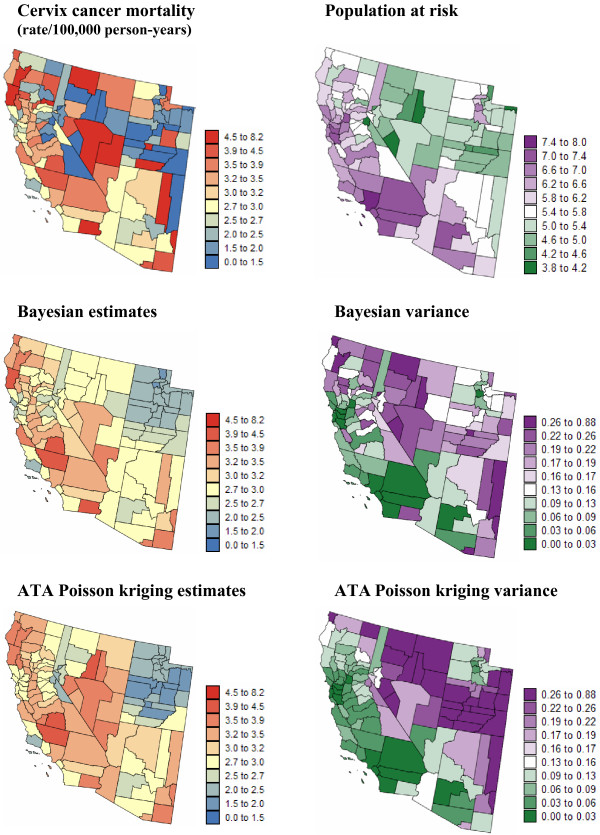
**Maps of age-adjusted cervix cancer mortality rates and the risk estimated using Bayesian and geostatistical methods**. The fill color in the left column maps represents the age-adjusted mortality rate per 100,000 person-years recorded over the period 1970–1994 (top graph) or the risk estimated using a Bayesian approach (BYM model) or ATA Poisson kriging. The class boundaries correspond to the deciles of the histogram of original rates. The corresponding prediction variance is mapped in the right column, along with the population at risk that was back-calculated from the rate and count data (lognormal scale).

In addition to mortality rates, the population at risk is available at the county level and over a grid of 25 km^2 ^cells discretizing each Region; see [25] for a description of these datasets. Figures 1 and 2 (top graphs) show county maps of age-adjusted mortality rates and population at risk, for lung cancer in Region 1 and cervix cancer in Region 2. The population-weighted average of mortality rates is 23.7 per 100,000 person-years for lung cancer and 2.85 per 100,000 person-years for cervix cancer.

### Simulated data sets

An objective assessment of the performance of the geostatistical and Bayesian disease mapping techniques requires knowledge of the "true" underlying risk maps, which are unknown in practice. Simulation provides a way to generate multiple realizations of the spatial distribution of cancer mortality rates under specific scenarios for the underlying risk and population sizes. Predicted risks can then be compared to the risk maps used in the simulation. For both lung and cervix cancers, *L *= 100 maps of county-level mortality rates {z^(l)^(*v*_*α*_), *α *= 1,..., N; l = 1,..., L} were generated in a previous study [25] using a two-step procedure: 1) county-level mortality risks were computed as population-weighted averages of continuous risk maps created by sequential Gaussian simulation, and 2) the number of death within each county was simulated by random drawing from a Poisson distribution characterized by the county-level risk and population. The present comparison study used the odd-numbered realizations (i.e. realization #1,3,5,...,99) for each set.

### Area-to-Area (ATA) Poisson kriging

The geostatistical methodology for the estimation of risk values from empirical frequencies, and the creation of isopleth maps, is described in details in Goovaerts [25]. This section provides a brief recall of the approach

For a given number *N *of geographical units *v*_*α *_(e.g. counties), denote the observed mortality rates (areal data) as *z*(*v*_*α*_) = *d*(*v*_*α*_)/*n*(*v*_*α*_), where *d*(*v*_*α*_) is the number of recorded mortality cases and *n*(*v*_*α*_) is the size of the population at risk. The noise-filtered mortality rate for a given area *v*_*α*_, called mortality risk, is estimated as a linear combination of the kernel rate *z*(*v*_*α*_) and the rates observed in (K-1) neighboring entities *v*_i_:

$${\hat{r}}_{PK}(v_{\alpha })=\sum_{i=1}^{K}\lambda_{i}(v_{\alpha })z(v_{i})$$

Unlike traditional kriging estimators, Poisson kriging is a non-exact interpolator since it does not return the observed rates: ${\hat{r}}_{PK}$ (*v*_*α*_) ≠ *z*(*v*_*α*_). The weights *λ*_*i*_(*v*_*α*_) assigned to the *K *rates are computed by solving the following system of linear equations; known as "Poisson kriging" system:

$$\begin{array}{l} \begin{array}{cc} \sum_{j=1}^{K}\lambda_{j}(v_{\alpha })\left[{\bar{C}}_{R}(v_{i},v_{j})+\delta_{ij}\frac{m^{*}}{n(v_{i})}\right]+\mu (v_{\alpha })={\bar{C}}_{R}(v_{i},v_{\alpha }) & i=1,...,K \end{array}\\ \sum_{j=1}^{K}\lambda_{j}(v_{\alpha })=1. \end{array}$$

where *μ*(*v*_*α*_) is the Lagrange parameter, m* is the population-weighted mean of the *N *rates, and *δ*_*ij *_= 1 if *v*_i _= *v*_j _and 0 otherwise. The "error variance" term, m*/*n*(*v*_i_), derives directly from the Poisson distribution for the disease count variable *D*(*v*_i_). This term, which corresponds to the difference between the variances of the risk and rate variables (Equation 5 in [22]), leads to smaller weights for less reliable data (i.e. rates measured over smaller populations). In addition to the population size, the kriging system accounts for the spatial correlation among geographical units through the area-to-area covariance terms ${\bar{C}}_{R}$(*v*_*i*_,*v*_*j*_) = Cov{*Z*(*v*_i_), *Z*(*v*_j_)} and ${\bar{C}}_{R}$(*v*_*i*_, *v*_*α*_). Those covariances are numerically approximated by averaging the point-support covariance *C*(**h**) computed between any two locations discretizing the areas *v*_i _and *v*_j_:

$${\bar{C}}_{R}(v_{i},v_{j})=\frac{1}{\sum_{s=1}^{P_{i}}\sum_{s^{\prime }=1}^{P_{j}}w_{ss^{\prime }}}\sum_{s=1}^{P_{i}}\sum_{s^{\prime }=1}^{P_{j}}w_{ss^{\prime }}C(u_{s},u_{s^{\prime }})$$

where *P*_*i *_and *P*_*j *_are the number of points used to discretize the two areas *v*_i _and *v*_j_, respectively. The weights *w*_*ss' *_are computed as the product of population sizes within the square cells centred on the discretizing point **u**_s _and **u**_s'_:

$$\begin{array}{lllll} w_{ss^{\prime }}=n(u_{s})\times (u_{s^{\prime }}) & \text{with} & \sum_{s=1}^{P_{i}}n(u_{s})=n(v_{i}) & \text{and} & \sum_{s^{\prime }=1}^{P_{j}}n(u_{s^{\prime }})=n(v_{j}) \end{array}$$

In this study the discretizing points were identified with the nodes of a 5 km grid, yielding a total of 9 to 69 points per county in Indiana, and 11 to 2,082 discretizing points for the West Coast counties. When the geographical units differ by several orders of magnitude like in Region 2, it is not computationally efficient to use the same discretizing level for each unit. One solution is to use flexible discretizing grids that ensure a constant number of discretizing points within each unit. For example, in TerraSeer's STIS software [29] a given number of discretization points is distributed uniformly within each polygon according to a stratified random design.

The point-support covariance of the risk C(**h**), or equivalently the point-support semivariogram *γ*(**h**), cannot be estimated directly from the observed rates, since only areal data are available. Thus, only the regularized semivariogram of the risk can be estimated as:

$${\hat{\gamma }}_{R}(h)=\frac{1}{2\sum_{\alpha ,\beta }^{N(h)}\frac{n(v_{\alpha })n(v_{\beta })}{n(v_{\alpha })+n(v_{\beta })}}\sum_{\alpha ,\beta }^{N(h)}\left\{\frac{n(v_{\alpha })n(v_{\beta })}{n(v_{\alpha })+n(v_{\beta })}{\left[z(v_{\alpha })-z(v_{\beta })\right]}^{2}-m^{*}\right\}$$

where N(**h**) is the number of pairs of areas (*v*_*α*_, *v*_*β*_) whose population-weighted centroids are separated by the vector **h**. The different spatial increments [*z*(*v*_*α*_)-*z*(*v*_*β*_)]^2 ^are weighted by a function of their respective population sizes, n(*v*_*α*_)n(*v*_*β*_)/[n(*v*_*α*_)+n(*v*_*β*_)], a term which is inversely proportional to their standard deviations. More importance is thus given to the more reliable data pairs (i.e. smaller standard deviations). Derivation of a point-support semivariogram from the model fitted to the experimental semivariogram ${\hat{\gamma }}_{R}$(**h**) computed from areal data is called "deconvolution", an operation that has been the topic of much research [30-33]. In this paper, we adopted the iterative procedure introduced for area-to-area Poisson kriging [25] whereby one seeks the point-support model that, once regularized, is the closest to the model fitted to areal data; see [33] for a detailed presentation of the algorithm and demonstration of its performances in simulation studies.

The uncertainty about the cancer mortality risk prevailing within the geographical unit *v*_*α *_can be modeled using the conditional cumulative distribution function (ccdf) of the risk variable *R*(*v*_*α*_). Under the assumption of normality of the prediction errors, that ccdf is defined as:

$$F(v_{\alpha };r|(K))=\text{Prob}\{R(v_{\alpha })\leq r|(K)\}=G\left(\frac{r-\hat{r}(v_{\alpha })}{\sigma (v_{\alpha })}\right)$$

*G*(·) is the cumulative distribution function of the standard normal random variable, and *σ*(*v*_*α*_) is the square root of the kriging variance estimated as:

$$\sigma^{2}(v_{\alpha })={\bar{C}}_{R}(v_{\alpha },v_{\alpha })-\sum_{i=1}^{K}\lambda_{i}{\bar{C}}_{R}(v_{i},v_{\alpha })-\mu (v_{\alpha })$$

where ${\bar{C}}_{R}$(*v*_*α*_, *v*_*α*_) is the within-area covariance that is computed according to Equation (3) with *v*_i _= *v*_j _= *v*_*α*_. This term will increase with the size of the area, leading to larger kriging variance when the risk is estimated for large geographical units. The notation "|(*K*)" expresses conditioning to the local information, say, *K *neighboring observed rates.

### Point Poisson kriging

Assimilating each unit *v*_*α *_to its population-weighted centroid **u**_*α *_greatly simplifies the implementation of the geostatistical methodology since it eliminates the need to discretize geographical units or perform a semivariogram deconvolution. Besides the gain in computational speed, the centroids-based (i.e. point) Poisson kriging can be accomplished using the public-domain executable **poisson-kriging.exe **described in Goovaerts [22]. This simplified version of Poisson kriging is included in the comparison study to: 1) quantify the potential loss in accuracy and precision resulting from such an approximation, and 2) apply Poisson kriging using the same point support as the BYM model.

### Bayesian model

Clayton and Kaldor [34] first introduced empirical Bayesian inference for relative risks. It was later extended to a fully Bayesian setting by Besag *et al*. [5]. The BYM model is one of the most popular hierarchical Bayesian models. This model incorporates random effects due to unstructured and spatially structured heterogeneity into the log-linear model for the relative risk. The inclusion of these random effects allows smoothing relative risks at global and local levels. For a detailed description of this model, the readers are referred to Lawson *et al*. [35] and Wakefield *et al*. [36]. The three stages of the BYM model, as described in Lawson *et al*. [35], are briefly reviewed in this section.

#### First-stage model

For rare diseases such as cancers, the observed number of mortality cases (*y*_*i*_) in a geographic unit *i *is assumed to follow a Poisson distribution with mean *e*_*i*_*θ*_*i*_:

*y*_*i *_~ *Poisson*(*e*_*i*_*θ*_*i*_)

where *e*_*i *_denotes the age-adjusted expected number of cases in the *i*^*th *^geographic unit, and *θ*_*i *_is the "true" but unknown relative risk in that unit. Besag *et al *[5] proposed the following model for the log relative risk:

log(*θ*_*i*_) = *μ *+ *v*_*i *_+ *u*_*i*_

where the intercept *μ *represents the global mean, and the terms *v*_*i *_and *u*_*i *_are random effects modeling the unstructured and spatially structured heterogeneity, respectively.

#### Second-stage model

Within the Bayesian framework, a prior distribution for the random effects and intercept term needs to be specified. In this paper the intercept was assigned improper flat prior. The uncorrelated component *v*_*i *_does not depend on geographic location and is assumed to follow a normal distribution with zero mean and a common variance (precision parameter) *τ*_*v*_^2^:

$$v_{i}\sim N(0,\tau_{v}^{2})$$

The random effect associated with spatial autocorrelation, *u*_*i*_, is defined according to the conditional autoregressive (CAR) Gaussian prior model [5]:

$$u_{i}|u_{j,j\neq i},\tau_{u}^{2}\sim N\left(\frac{\sum_{j}w_{ij}u_{j}}{\sum_{j}w_{ij}},\frac{\tau_{u}^{2}}{\sum_{j}w_{ij}}\right)$$

where the prior mean of each *u*_*i *_is defined as a weighted average of the other *u*_*j*_, j≠i. The weights *w*_*ij *_define the relationship between the area *i *and its neighbors *j*. We adopted the common first order binary weighting scheme where *w*_*ij*_*=*1 if areas *i *and *j *share a common border, and *w*_*ij *_= 0 otherwise. The precision parameter *τ*_*u*_^2 ^controls the amount of variability for the random effect.

#### Third-stage model

A fully Bayesian model specification is completed by adding hyper-prior distributions on the precision parameters *τ*_*v*_^2 ^and *τ*_*u*_^2^. With no prior estimation for precisions of the random effects, distributions with large variance (i.e. vague hyperpriors) are recommended. Following Kelsall and Wakefield's discussion [37] we adopted the Gamma distribution Γ(0.5, 0.0005) which yields a probability of 99% that the precision lies between 0.16 and 6635, with most of the probability concentrated towards 0 [9]. This prior choice is less informative and allows the likelihood data to dominate the prior information; hence, it will have minimum effect on the inference of relative risks.

#### Implementation of the model

As mentioned in the introduction, Bayesian inference of model parameters is not feasible through mathematically closed form. Instead, it requires computer intensive MCMC simulations, such as Gibbs sampler, which became affordable with recent increase in computational power. The BYM model for each data set (lung and cervix cancers) was fitted using WinBUGS version 1.4 [7] that implements the Markov Chain Monte Carlo (MCMC) methods [38]. The model was simulated using two independent chains starting with dispersed initial values. Convergence of the relative risks for the two chains was confirmed by graphing their traces, as well as computing the Gelman-Rubin (G-R) statistic and Monte Carlo errors (<5% of the posterior standard deviation). After a burn-in of 15,000 iterations, the following 10,000 iterations were sampled from the two chains, yielding a set of 20,000 values to approximate the posterior distribution of the relative risk for each geographical unit (i.e. county). For comparison with the Poisson kriging absolute risk estimate, each simulated relative risk value was multiplied by the population-weighted average of rates. The distributions of 20,000 sampled values were post-processed to derive summary statistics, such as mean, variance or the probability of exceeding specific risk thresholds.

## Results and Discussion

### Analysis of lung and cervix cancer data

**Figure 3:**
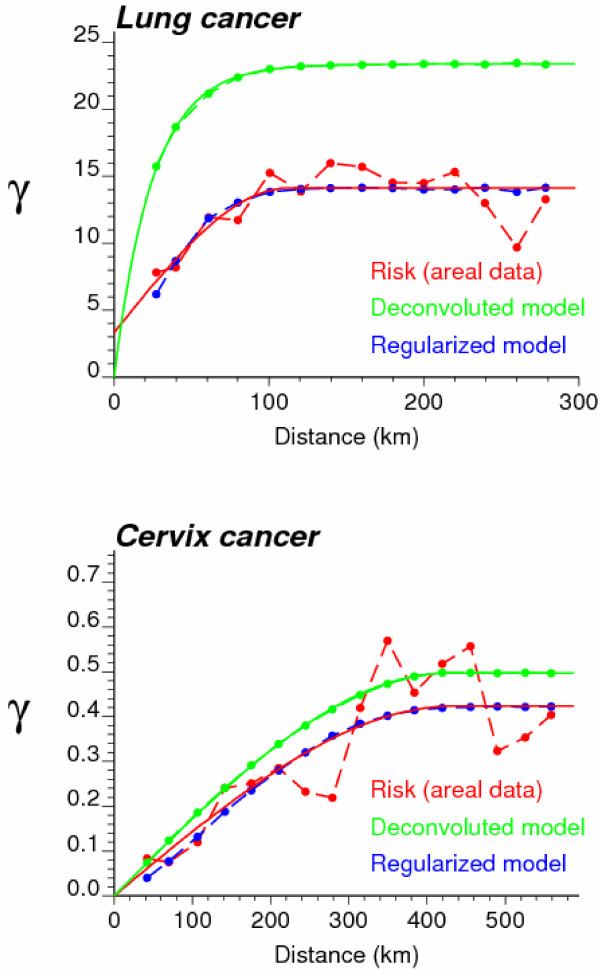
**Semivariogram models used by Poisson kriging for mapping lung and cervix cancer mortality**. The experimental semivariogram of the risk (red curve) is estimated from county-level rate data, and its deconvolution yields the point-support model (green curve) required by area-to-area Poisson kriging. The regularization of the point support model yields a curve (blue line) that is very close to the experimental one.

Mortality risks for lung and cervix cancers were estimated from the rates displayed at the top of Figures 1 and 2 using a Bayesian approach (BYM model) and ATA Poisson kriging. To facilitate the comparison between the two models, the same set of neighbors (i.e. adjacent counties) was used in both cases. Poisson kriging was performed using the point-support semivariogram models displayed in Figure 3 (green curve). These models were derived by deconvolution of county-level models fitted to omnidirectional semivariograms (Equation 5) of rates (Figure 3, red curve). As expected, the point-support semivariogram model has a higher sill since the punctual process has a larger variance than its aggregated form. The blue curve represents the theoretically regularized semivariogram model that is close to the one fitted to experimental values, which validates the consistency of the deconvolution; see [25,33] for a detailed discussion of the deconvolution procedure. In addition to county-level risk estimates, the geostatistical and Bayesian models provide the estimation variance that is mapped at the bottom of Figures 1 and 2 (right column). Summary statistics for both sets of maps are listed in Table 1.

**Table 1:** Summary statistics for estimates of lung and cervix cancer mortality.

Estimators	Lung cancer			Cervix cancer		
	Mean	Variance	Min-max	Mean	Variance	Min-Max

Observed rates	21.19	18.48	9.071–31.79	2.851	2.446	0.000–8.138
ATA Poisson kriging (PK) estimate	21.51	11.31	13.07–31.55	2.880	0.320	1.800–4.044
Point Poisson kriging (PK) estimate	21.52	11.13	13.33–31.61	2.897	0.338	1.811–4.061
BYM model estimate	21.38	11.35	13.96–31.63	2.889	0.200	1.915–4.074
ATA Poisson kriging (PK) variance	3.122	2.009	0.238–7.582	0.187	0.040	0.003–0.881
Point Poisson kriging (PK) variance	3.275	2.367	0.242–7.731	0.211	0.043	0.003–0.948
BYM model variance	3.224	3.288	0.306–12.05	0.154	0.009	0.004–0.509

Correlation PK vs ATA PK estimate	0.997			0.989		
Correlation BYM vs ATA PK estimate	0.988			0.845		
Correlation PK vs ATA PK variance	0.989			0.986		
Correlation BYM vs ATA PK variance	0.908			0.549		

Mean, variance and range of cancer mortality rates per 100,000 person-years estimated at the county level using Poisson kriging (point and ATA) and BYM model. Statistics for the kriging variance and variance of Bayesian empirical distribution are listed below.

#### Mortality risk estimates

**Figure 4:**
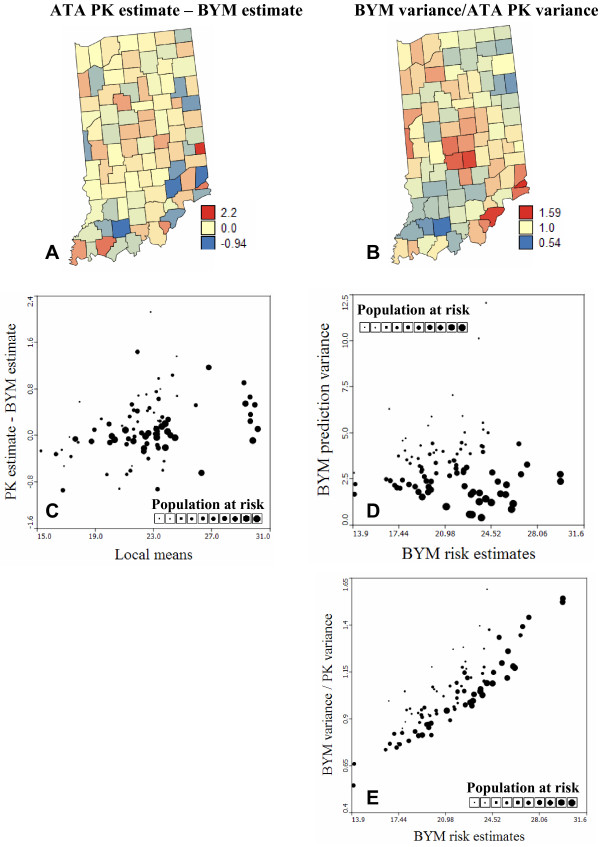
**Differences between the lung cancer mortality risk and prediction variance computed by the Bayesian and geostatistical methods**. Maps (A, B) highlight the counties where the Bayesian and geostatistical methods differ the most in terms of risk estimates (absolute differences) and prediction variance (relative differences). A diverging color scheme with three breaks (mid-point = 0 or 1) was chosen; counties with values in between the breaks receive a blend of the two break colors. (C) ATA Poisson kriging yields larger risk estimates than BYM model in high-valued areas, while lower risks are predicted in low-valued areas. (D, E) The lognormal hypothesis underlying the BYM model leads to larger prediction variance for larger risk estimates, once the effect of the population at risk is accounted for through the division by the kriging variance. In all scatterplots, the size of the dots is proportional to the population at risk.

The two risk maps of Region 1 are very similar (correlation = 0.988); see Figure 1 (left column). Differences between both models decrease as the population at risk increases, which is expected since the mortality rates computed from large populations tend to be more reliable and are little impacted by the smoothing procedure. This similarity for large populations at risk is illustrated in Figure 4C: densely populated counties depicted by bigger dots in the scatterplot show very small differences between ATA Poisson kriging and BYM estimates. The horizontal axis represents the local mean of rates estimated as the population-weighted average of rates recorded in adjacent counties. This scatterplot reveals the tendency for geostatistical risk estimates to exceed Bayesian estimates for sparsely populated counties located in high-valued areas (high local means). Conversely, geostatistical estimates are smaller than Bayesian estimates in low-valued areas. This effect is however of small magnitude for this geography, and both maps of risk estimates have fairly similar mean and variance (Table 1). Point and ATA Poisson kriging yields very similar risk estimates (correlation = 0.997).

**Figure 5:**
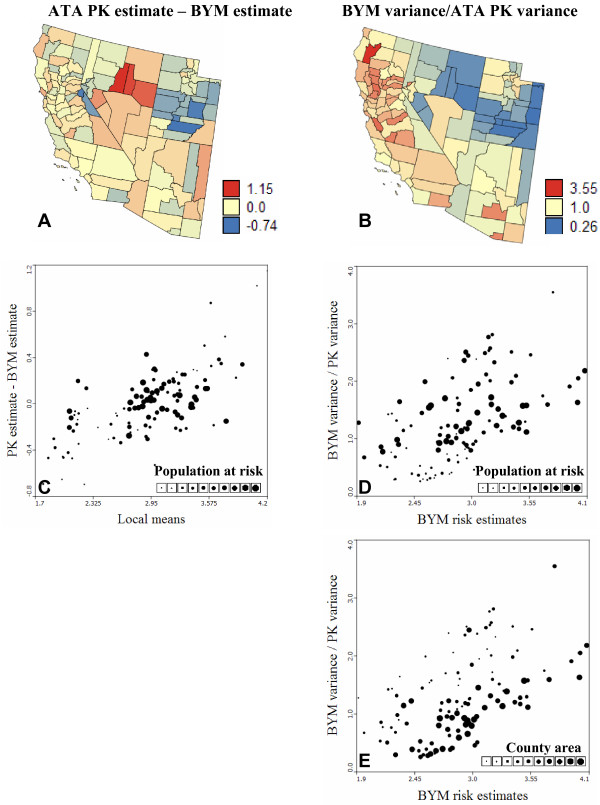
**Differences between the cervix cancer mortality risk and prediction variance computed by the Bayesian and geostatistical methods**. Maps (A, B) highlight the counties where the Bayesian and geostatistical methods differ the most in terms of risk estimates (absolute differences) and prediction variance (relative differences). A diverging color scheme with three breaks (mid-point = 0 or 1) was chosen; counties with values in between the breaks receive a blend of the two break colors. (C) ATA Poisson kriging yields larger risk estimates than BYM model in high-valued areas, while lower risks are predicted in low-valued areas. (D, E) The lognormal hypothesis underlying the BYM model leads to larger prediction variance for larger risk estimates, while the ATA Poisson kriging variance increases in counties with small population at risk and large areas. The size of the dots in scatterplots is proportional either to the size of the population at risk or the area of each county.

Discrepancies between risk maps become more noticeable for the less frequent cervix cancer (Figure 2, left column). The correlation between BYM and geostatistical estimates is smaller (0.845) and the BYM risk map is clearly smoother: the variance of Bayesian estimates is 40% smaller than the variance of kriging estimates (Table 1). Due to the NE-SW trend in cervix cancer mortality, the local mean greatly varies across Region 2 and displays a clear relationship with the sign and magnitude of the difference between BYM and geostatistical estimates (correlation = 0.71); see Figure 5C. Poisson kriging yields smaller risk estimates across Utah, while risks estimated across Nevada are higher than for the BYM model (Figure 5A). Like in Region 1, discrepancies between methods increase in sparsely populated counties, which also contributes to the large differences observed in the less densely populated states of Utah and Nevada. The correlation between point and ATA PK risk estimates is slightly smaller than in Region 1 (0.989 vs. 0.997), yet it is still very strong.

#### Prediction variance

The Bayesian and geostatistical models differ much more in terms of prediction variance than estimated risk; see Figures 1 and 2 (right column). For both models the prediction variance increases as the population at risk, hence the reliability of raw rates, decreases. Because it accounts for the shape and size of counties, the ATA Poisson kriging variance also increases for larger spatial supports, i.e. counties of large extent. On the other hand, the lognormal hypothesis causes the BYM prediction variance to increase with the estimated risk. In Region 1 this latter effect is not obvious at first glance because it is masked by the impact of population size (Figure 4D). The proportional effect becomes striking when considering the ratio of the BYM variance versus the ATA Poisson kriging variance, which filters the effect of population size (Figure 4E). The correlation between this ratio and the BYM risk estimate is 0.85, indicating that the difference between the two types of prediction variance is largely controlled by the magnitude of Bayesian risk estimates. Figure 4E also indicates that conditionally to a range of BYM estimates, the BYM variance decreases faster than the ATA Poisson kriging variance as the population size, depicted by the size of the dots in the scatterplot, increases.

The smaller correlation between BYM and geostatistical estimates in Region 2, combined with the wide range of sizes and shapes of counties, enhances the differences between the prediction variance maps (Figure 2, right column). The correlation between the Bayesian and geostatistical prediction variances is only 0.549. The map of the ratio of the BYM variance versus the ATA Poisson kriging variance bears a lot of similarity with the map of risk estimates. The BYM prediction variance is smaller than the Poisson kriging variance in low risk areas, such as Utah, while the opposite trend is observed in the high risk areas along the coast (Figure 5B). The relationship between the variance ratio and the BYM risk estimate is not as strong as in Region 1: the correlation is 0.53 instead of 0.85 for Indiana. This weaker correlation reflects the impact of the size of geographical units on the kriging variance which is more pronounced for this heterogeneous county geography (Figure 5D). For example, Figure 5E illustrates the larger ATA Poisson kriging variance, hence smaller variance ratio, for counties of large size depicted by bigger dots in the scatterplot.

The spatial support (i.e. point versus area) has a much smaller impact on the prediction variance than the statistical methodology (i.e. geostatistical versus Bayesian models); see Table 1 (last two rows). For both regions the correlation between ATA and point kriging variances is strong: 0.986–0.989. Ignoring the spatial support of county-level data however leads, on average, to larger kriging variances: 3.275 vs 3.122 for Region 1 and 0.211 vs 0.187 for Region 2. This result is consistent with the "change-of-support" theory that predicts larger error variances for point versus areal estimation. The relative magnitude of the overestimation by point kriging is the largest for Region 2 that includes very large counties and has a wide range of county sizes and shapes.

#### Decision-making

**Figure 6:**
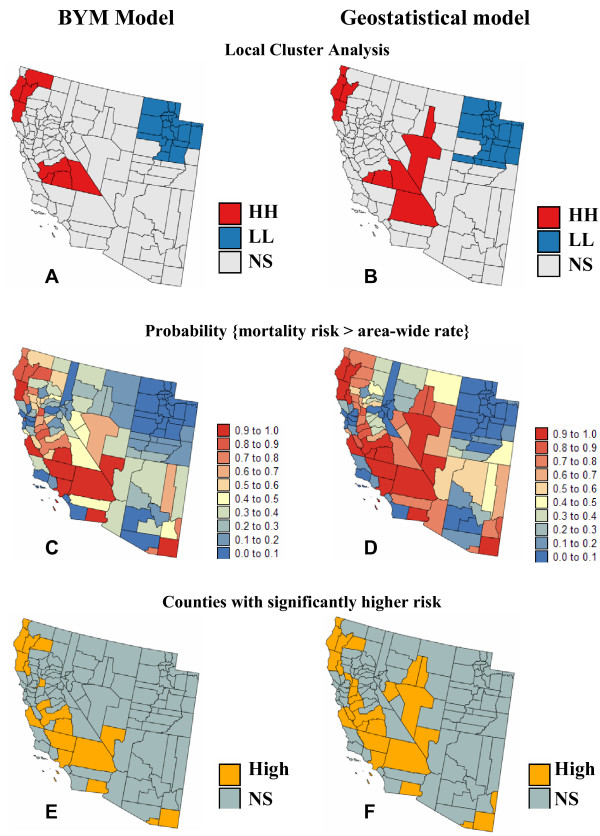
**Impact of modeling approach on the results of local cluster analysis and detection of counties with significantly higher mortality risk**. The fill color in top maps (A, B) represents the classification of counties into significant low-low (LL) or high-high (HH) clusters based on a local cluster analysis of Bayesian and geostatistical (ATA PK) risk estimates. Light gray indicates counties that are not significant at the level *α *= 0.05; the *p*-values were corrected for multiple testing using the Simes adjustment. (C, D) The probability that the county-level mortality risk exceeds the area-wide rate (i.e. population-weighted average for all 118 counties) is computed for both the BYM and geostatistical models. (E, F) Counties with a probability larger than 0.75 are flagged as having significantly higher risk and depicted in orange.

Mapping cancer risk is a preliminary step towards further analysis that might highlight areas where causative exposures change through geographic space, the presence of local populations with distinct cancer incidences, or the impact of different cancer control methods. Figure 6 illustrates the impact of the modeling approach on the detection of cancer clusters and the identification of counties with significantly higher mortality risk in Region 2.

The local Moran test to evaluate local clustering or spatial autocorrelation [39] was applied to the map of Bayesian and geostatistical risk estimates. The use of PK instead of BYM estimates increases the size of the central cluster of high mortality risk and the Utah cluster of low risk, compare Figure 6A and 6B. This difference results directly from the geostatistical prediction of lower risks in low-valued areas (i.e. Utah) and higher risks in high-valued areas (i.e. Nevada), recall Figure 5A. Beware that the use of smoothed rates in local cluster analysis (LCA) ignores the uncertainty attached to estimated risk and tends to create artificially large clusters. A geostatistical-based simulation procedure that propagates the rate uncertainty through LCA, as described in [24], would be more appropriate.

To depict the uncertainty attached to risk maps, several authors recommend mapping the 95^th ^percentile range of the posterior distribution of risk values or the probability that the risk in each entity exceeds a specific threshold of interest [9,40]. This probability is readily derived from the PK estimate and variance using the analytical expression (6) or it can be computed from the empirical BYM posterior distribution. As an example, the population-weighted average of cervix cancer mortality rates (i.e. area-wide rate) was used as threshold of interest. The corresponding probability maps displayed in Figures 6C &6D capture the features of both the risk and prediction variance maps. In particular, note the larger probability of exceedence computed for Nevada using the kriging results. Richardson *et al*. [14] proposed to use this type of probability to decide whether an area should be classified as having an excess risk of cancer. Following their decision rule that the probability must exceed 0.75 in order for an area to be classified as having increased risk yields the classification maps at the bottom of Figure 6. The geostatistical model leads to declaring substantially more counties as having elevated risk: 28 instead of the subset of 18 for the BYM model. Four of these additional ten counties are located in Nevada, a result in agreement with the local cluster analysis. Note that the decision rule considered in this example ignores the multiple testing (or multiple comparison) problem caused by the repeated use of statistical tests; see [41] for a discussion on correction for multiple testing.

### Simulation studies

Figures 1, 2, 3, 4, 5, 6 supported an empirical comparison of results obtained using the BYM model and Poisson kriging. The smoothing of BYM risk estimates and smaller proportion of significant raised-risk areas agree with previous studies that showed Bayesian disease-mapping models to be conservative [14]. However, since the "true" mortality risk is unknown, one cannot conclude that one approach outperforms the other. To investigate the performance of Bayesian and geostatistical disease-mapping models for recovering the "true" risk surfaces, in particular the ability to detect risk-raised areas, a similar analysis was conducted on 50 simulated rate maps. Predicted risks were then compared to the risk maps used in the simulation.

**Figure 7:**
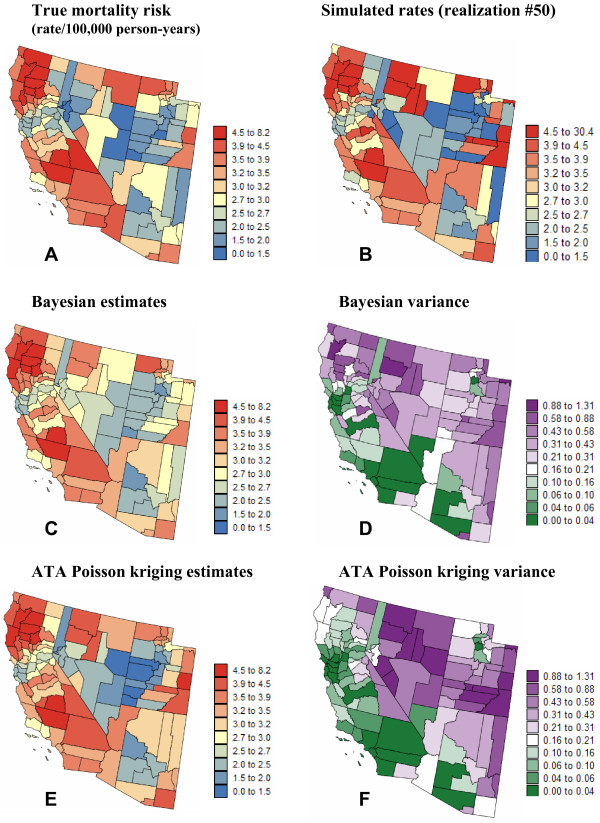
**Simulated cervix cancer risk map and the results of the Bayesian and geostatistical analysis**. The number of cases for each county was simulated by random sampling of a Poisson distribution that is defined by the white female population map of Figure 2 and a "true" risk map (A) generated using sequential Gaussian simulation. (C, E) Risk maps estimated from simulated rates using a Bayesian approach or ATA Poisson kriging. The class boundaries correspond to the deciles of the histogram of original rates. (D, F) Prediction variance associated with the Bayesian and geostatistical risk estimates.

For example, Figure 7A shows the "true" risk map that was combined with the population map of Figure 2 to generate the 50^th ^simulated rate map in Region 2 (Figure 7B). To be more specific, the number of cases for each county was simulated by random sampling of a Poisson distribution whose mean parameter is the product of the mortality risk by the population. The division of the simulated death counts by the county population leads the set of simulated mortality rates; see [25] for more details. The noise caused by the small number problem is particularly apparent for sparsely populated counties. For example, a couple of counties in the North central part of the simulated map display high mortality rates, while the underlying risk value is low.

**Figure 8:**
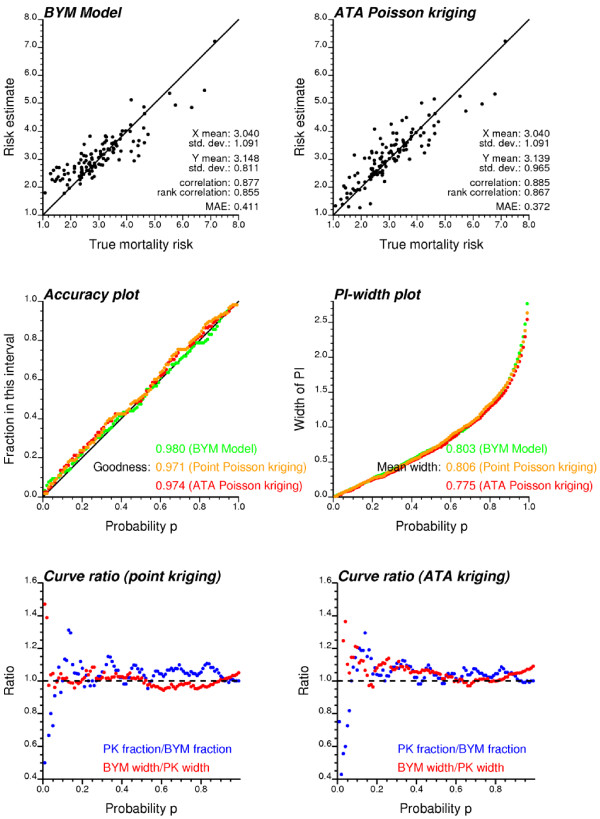
**Impact of modeling approach on the prediction accuracy and the precision of the probability intervals (realization #50)**. Fifty realizations of the spatial distribution of cervix cancer mortality rates in Region 2 were simulated and then analyzed using a Bayesian (BYM model) and a geostatistical (point and area-to-area Poisson kriging) approach. Results for the 50^th ^realization are presented. Top scatterplots (A, B) illustrate that the geostatistical risk estimates are better correlated with true risk values (smaller Mean Absolute Error of prediction, MAE) than the Bayesian estimates. Plot of the fraction of true mortality risk values falling within probability intervals (C), and the width of these intervals versus the probability p (D). The goodness statistic measures the similarity between the expected and observed fractions in the accuracy plots (best if closer to 1). Narrower probability intervals (i.e. smaller mean widths) indicate more precise models of uncertainty. (E) Ratio of accuracy and PI-width curves; whenever both ratios exceed one (black dashed line), the geostatistical PI is narrower than the Bayesian PI, while including a larger fraction of true values.

The Bayesian and geostatistical analysis of the simulated rate map confirms the conclusions drawn from the analysis of real cancer mortality rates in Figure 2. The BYM risk map (Figure 7C, std deviation = 0.811) is smoother than the map created using ATA Poisson kriging (Figure 7E, std deviation = 0.965). This effect is particularly obvious for Utah where the geostatistical risk map exhibits a strong contrast between the East and West sides of the state, while the BYM model is much more flat. The smoothing of the BYM model leads to an overestimation of low risk values (Figures 8A &8B). Discrepancies between the Bayesian and geostatistical models are even larger for the prediction variance (Figures 7D &7F). For example, the proportional effect of the BYM model results in larger prediction variance in Northern California, where risk estimates are high, while the ATA Poisson kriging variance is smaller in these densely populated counties.

**Figure 9:**
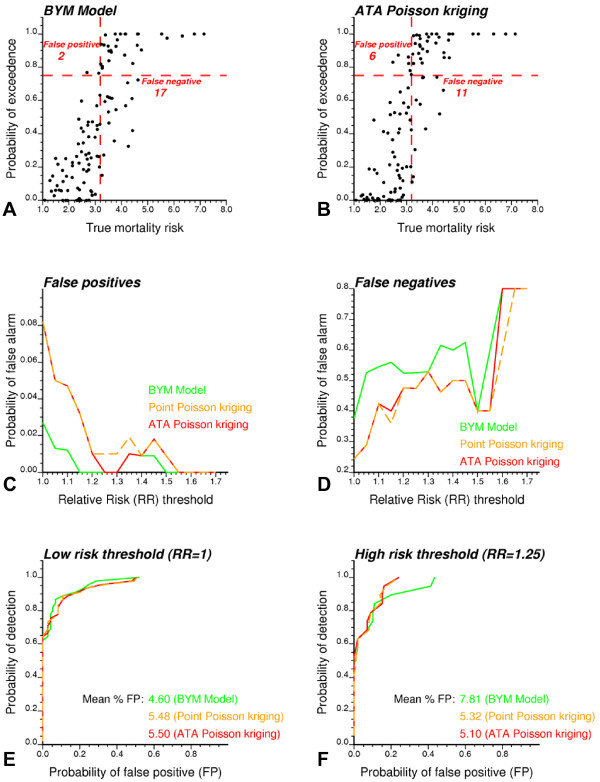
**Impact of modeling approach and risk threshold on the proportion of counties wrongly classified as having low or high cancer mortality risk (realization #50)**. Fifty realizations of the spatial distribution of cervix cancer mortality rates in Region 2 were simulated and then analyzed using a Bayesian (BYM model) and a geostatistical (point and area-to-area Poisson kriging) approach. Results for the 50^th ^realization are presented. If a county has at least a 0.75 probability to exceed a risk threshold equal to the area-wide rate (3.186), it is flagged as having significantly higher risk, resulting in potential false positives and negatives based on the actual mortality risk (A, B). The proportion of false alarms is computed for a range of risk thresholds expressed as multiples of the area-wide rate (C, D). Receiver Operating Characteristic (ROC) curves plot the probability of false positive versus the probability of detection for two different thresholds (E, F). The average percentage of false positives (FP) is smaller for the Bayesian approach relatively to Poisson kriging for the low threshold but larger for the high threshold.

These maps are now compared to the reference risk map, and results for realization #50 in Region 2 are illustrated in Figures 8 and 9. Results averaged over all 50 realizations for the two regions are reported in Tables 2, 3, 4, 5, 6, 7. To investigate the impact of the search strategy, ATA Poisson kriging was conducted using either adjacent counties (same neighbors as BYM model) or the 32 closest counties in terms of distance between population-weighted centroids. Straightforward empirical Bayesian smoothers were also applied to quantify the benefit of more complex Bayesian and geostatistical models.

**Table 2:** Performance comparison of Bayesian and geostatistical estimators: mean error of prediction.

Estimators	Lung cancer		Cervix cancer	
**Arithmetical average**	Average	% best result	Average	% best result

Global Empirical Bayes	-0.031	22	0.053	18
Local Empirical Bayes	0.012	14	**0.006**	28
BYM Model	0.010	12	**0.006**	16
Point Poisson kriging (adjacent counties)	0.009	14	0.019	22
ATA Poisson kriging (adjacent counties)	0.014	20	0.023	8
ATA Poisson kriging (32 neighbors)	**-0.001**	18	0.040	8

**Population-weighted average**				
Global Empirical Bayes	-0.026	26	0.012	32
Local Empirical Bayes	-0.011	22	0.001	32
BYM Model	**-0.009**	18	**0.001**	12
Point Poisson kriging (adjacent counties)	-0.011	16	**0.001**	10
ATA Poisson kriging (adjacent counties)	-0.011	4	**0.001**	8
ATA Poisson kriging (32 neighbors)	-0.016	14	0.002	6

Results obtained on average (arithmetical and population-weighted) over 50 realizations generated for Regions 1 and 2. Poisson kriging was conducted using either adjacent counties (same neighbors as BYM model) or the 32 closest counties in terms of distance between population-weighted centroids. ATA kriging accounts for the shape and size of the counties in the analysis. Straightforward empirical Bayesian smoothers were also applied. Bold numbers refer to best performances. The second column gives the percentage of realizations where the particular method yields the smallest prediction error.

**Table 3:** Performance comparison of Bayesian and geostatistical estimators: mean absolute error of prediction.

Estimators	Lung cancer (mean = 21.25)		Cervix cancer (mean = 2.993)	
**Arithmetical average**	Average	% best result	Average	% best result

Global Empirical Bayes	1.396	2	0.517	0
Local Empirical Bayes	1.380	0	0.458	0
BYM model	1.280	6	0.426	0
Point Poisson kriging (adjacent counties)	**1.243**	30	0.400	16
ATA Poisson kriging (adjacent counties)	1.246	32	0.397	14
ATA Poisson kriging (32 neighbors)	1.250	30	**0.380**	70

**Population-weighted average**				
Global Empirical Bayes	0.972	2	0.152	0
Local Empirical Bayes	0.980	2	0.145	0
BYM model	0.918	12	0.141	4
Point Poisson kriging (adjacent counties)	**0.903**	32	0.133	18
ATA Poisson kriging (adjacent counties)	0.909	24	**0.132**	28
ATA Poisson kriging (32 neighbors)	0.911	28	**0.132**	50

Results obtained on average (arithmetical and population-weighted) over 50 realizations generated for Regions 1 and 2. Poisson kriging was conducted using either adjacent counties (same neighbors as BYM model) or the 32 closest counties in terms of distance between population-weighted centroids. ATA kriging accounts for the shape and size of the counties in the analysis. Straightforward empirical Bayesian smoothers were also applied. Bold numbers refer to best performances. The second column gives the percentage of realizations where the particular method yields the smallest prediction error.

**Table 4:** Performance comparison of Bayesian and geostatistical estimators: Smoothing effect and prediction variance.

Estimators	Lung cancer		Cervix cancer	
	Dispersion variance	Prediction variance	Dispersion variance	Prediction variance

True risk values	9.817	-	1.153	-
Global Empirical Bayes	6.059	-	0.537	-
Local Empirical Bayes	7.905	-	1.063	-
BYM model	7.034	2.791	0.757	0.347
Point Poisson kriging (adjacent counties)	7.835	2.542	1.006	0.338
ATA Poisson kriging (adjacent counties)	7.563	2.363	0.977	0.287
ATA Poisson kriging (32 neighbors)	7.400	2.297	0.830	0.237

Results obtained on average over 50 realizations generated for Regions 1 and 2. Poisson kriging was conducted using either adjacent counties (same neighbors as BYM model) or the 32 closest counties in terms of distance between population-weighted centroids. ATA kriging accounts for the shape and size of the counties in the analysis. Straightforward empirical Bayesian smoothers were also applied. The dispersion variance measures the variability of the set of risk estimates, while the prediction variance quantifies the uncertainty attached to county-level risk estimates.

**Table 5:** Performance comparison of geostatistical and Bayesian estimators: goodness and precision of models of uncertainty.

Estimators	Lung cancer		Cervix cancer	
GOODNESS STATISTIC	Average	% best result	Average	% best result

BYM model	**0.949**	48	0.950	48
Point Poisson kriging (adjacent counties)	0.941	28	**0.951**	24
ATA Poisson kriging (adjacent counties)	0.922	14	0.939	16
ATA Poisson kriging (32 neighbors)	0.914	10	0.927	12

AVERAGE WIDTH OF PI				

BYM model	2.544	14	0.816	4
Point Poisson kriging (adjacent counties)	2.439	20	0.813	0
ATA Poisson kriging (adjacent counties)	2.348	12	0.745	0
ATA Poisson kriging (32 neighbors)	**2.313**	54	**0.691**	96

% ACCURATE AND PRECISE PI				

BYM model vs Point PK (adjacent counties)	9.35	6	12.73	8
BYM model vs ATA PK (adjacent counties)	7.53	4	6.22	2
BYM model vs ATA PK (32 neighbors)	7.35	4	4.30	4
Point PK (adjacent counties) vs BYM model	**34.87**	36	28.02	32
ATA PK (adjacent counties) vs BYM model	28.12	24	36.02	20
ATA PK (32 neighbors) vs BYM model	27.66	26	**40.28**	34

Results obtained on average over 50 realizations generated for Regions 1 and 2. Poisson kriging was conducted using either adjacent counties (same neighbors as BYM Model) or the 32 closest counties in terms of distance between population-weighted centroids. ATA kriging accounts for the shape and size of the counties in the analysis. The last statistic, based on the pair wise comparison of methods, reports the percentage of probability intervals (PI) that are jointly more accurate and precise. Bold numbers refer to best performances: goodness close to one, narrow probability intervals, and high percentage of accurate and precise PIs. The second column gives the percentage of realizations where the particular method yields the best results.

**Table 6:** Performance comparison of geostatistical and Bayesian estimators: discriminatory power of models of uncertainty.

Estimators	Lung cancer		Cervix cancer	
LOW RISK THRESHOLD (RR = 1)	Average	% best result	Average	% best result

BYM model	4.009	14	3.532	16
Point Poisson kriging (adjacent counties)	4.112	20	3.751	4
ATA Poisson kriging (adjacent counties)	**4.186**	36	3.915	38
ATA Poisson kriging (32 neighbors)	4.132	30	**3.995**	42

HIGH RISK THRESHOLD	RR = 1.1		RR = 1.25	

BYM model	7.037	6	5.859	2
Point Poisson kriging (adjacent counties)	7.842	12	6.300	0
ATA Poisson kriging (adjacent counties)	8.059	36	6.721	6
ATA Poisson kriging (32 neighbors)	**8.243**	46	**7.381**	92

Results obtained on average over 50 realizations generated for Regions 1 and 2. Poisson kriging was conducted using either adjacent counties (same neighbors as BYM Model) or the 32 closest counties in terms of distance between population-weighted centroids. ATA kriging accounts for the shape and size of the counties in the analysis. The reported statistic is the average probability of exceeding a risk threshold for counties with true risk above that threshold divided by the average probability for the remaining counties. A low (Relative Risk, RR = 1) and high risk threshold (RR = 1.1, RR = 1.25) were considered. Bold numbers refer to best performances: large probability ratio. The second column gives the percentage of realizations where the particular method yields the best results.

**Table 7:** Performance comparison of geostatistical and Bayesian estimators: proportion of false positives.

Estimators	Lung cancer		Cervix cancer	
LOW RISK THRESHOLD (RR = 1)	Average	% best result	Average	% best result

BYM model	0.071	30	0.077	12
Point Poisson kriging (adjacent counties)	**0.069**	20	0.066	14
ATA Poisson kriging (adjacent counties)	0.070	30	0.063	24
ATA Poisson kriging (32 neighbors)	0.072	20	**0.061**	50

HIGH RISK THRESHOLD	RR = 1.1		RR = 1.25	

BYM model	0.069	20	0.080	4
Point Poisson kriging (adjacent counties)	0.066	14	0.065	12
ATA Poisson kriging (adjacent counties)	**0.064**	36	0.064	32
ATA Poisson kriging (32 neighbors)	**0.064**	30	**0.060**	52

Results obtained on average over 50 realizations generated for Regions 1 and 2. Poisson kriging was conducted using either adjacent counties (same neighbors as BYM Model) or the 32 closest counties in terms of distance between population-weighted centroids. ATA kriging accounts for the shape and size of the counties in the analysis. The proportion of false positives is derived from Receiver Operating Characteristic (ROC) curves computed using a low (Relative Risk, RR = 1) and high risk threshold (RR = 1.1, RR = 1.25). Bold numbers refer to best performances: few false positives. The second column gives the percentage of realizations where the particular method yields the best results.

#### Bias and accuracy of prediction

The first two criteria are the mean error (ME) and mean absolute error (MAE) of prediction computed as:

$$\begin{array}{lll} ME=\frac{1}{W}\sum_{\alpha =1}^{N}\omega_{\alpha }\left[r_{P}^{*}(v_{\alpha })-r(v_{\alpha })\right] & \text{with} & \text{W}=\sum_{\alpha =1}^{N}\omega_{\alpha } \end{array}$$

$$\begin{array}{lll} MAE=\frac{1}{W}\sum_{\alpha =1}^{N}\omega_{\alpha }\left|r_{P}^{*}(v_{\alpha })-r(v_{\alpha })\right| & \text{with} & \text{W}=\sum_{\alpha =1}^{N}\omega_{\alpha } \end{array}$$

where *r*(*v*_*α*_) is the "true" simulated risk and $r_{P}^{*}$(*v*_*α*_) is the risk estimated using Bayesian or geostatistical smoothers. The prediction error for each of the N counties is either equally weighted (*ω*_*α *_= 1) or weighted according to the population size (*ω*_*α *_= n(*v*_*α*_)), in order to penalize more the errors that affect a larger population.

Table 2 indicates that, on average over 50 realizations, all prediction methods are unbiased regardless of the weighting scheme or the cancer frequency. Methods differ more in terms of the mean absolute error of prediction, in particular for the least frequent cervix cancer (Table 3). The empirical Bayesian smoothers are consistently outperformed by other methods, although the average magnitude of the error never exceeds 5% of the mean rate. The benefit of Poisson kriging over BYM model is fairly systematic since it leads to smaller MAE values 100% of the time for cervix and 92% for lung cancer (see Figures 8A &8B for an illustration). Differences between methods are smaller for population-weighted statistics, yet Poisson kriging still yields the smallest prediction errors 96% of the time for cervix and 84% for lung cancer. Point Poisson kriging performs as well as ATA Poisson kriging, in particular in Region 1 where all counties have similar size and shape.

#### Smoothing effect and prediction variance

The comparison of risk maps in Figures 1, 2 and 7 indicated the smaller dispersion variance (i.e. larger smoothing) of estimates calculated using the BYM model. Results averaged over all 50 realizations and summarized in Table 4 support this finding. The variance of the reference risk values, averaged over the five scenarios for the risk map, is 9.817 and 1.153 for lung and cervix cancers, respectively. As expected, the global empirical Bayes estimator generates the largest smoothing effect: the variance represents 47–62% of the reference risk variance. The BYM model is the second worse (66–77%). The variance of ATA Poisson kriging estimates is closer to the variance of the true risk values, in particular when fewer observations (i.e. adjacent counties versus 32 nearest counties) are used in the estimation (77–85%). The smoothing effect is smaller for point Poisson kriging, which is expected since point estimates typically vary more than areal estimates.

In addition to a risk estimate, the BYM model and Poisson kriging provide a measure of the uncertainty attached to this estimate in the form of a prediction variance. For each interpolation method, the prediction variance was averaged over all counties, and the mean variances over all 50 realizations are listed in Table 4. As expected, the Poisson kriging variance increases when the estimation is based on fewer observations (i.e. adjacent counties versus 32 nearest counties) or ignores the support of the data (i.e. point versus ATA kriging). Yet, it remains on average 10–17% smaller than the prediction variance calculated for the BYM model. According to this criterion, the geostatistical model of uncertainty is more precise than the Bayesian one.

#### Quality of the model of uncertainty

The prediction variance does not fully capture the uncertainty attached to risk estimates, in particular for the BYM model where the entire probability distribution in the form of a set of simulated risk values is available for each geographic unit. From the distribution of 20,000 BYM simulated values we computed, for each of the counties, a series of symmetric *p*-probability intervals (PI) bounded by the (1-*p*)/2 and (1+*p*)/2 quantiles of that distribution. For example, the 0.5-PI is bounded by the lower and upper quartiles. Similar PIs were computed analytically from the Poisson kriging estimate and variance, under the assumption of normality of the prediction errors (Equation 6).

A correct modeling of local uncertainty would entail that, for example, there is a 0.5-probability that the actual mortality risk falls into the 0.5-PI or, equivalently, that over the study area 50% of the 0.5-PI include the true risk value. These fractions are easily computed for simulation results since the true risk map is known. Following Deutsch [42], the agreement between observed, $p_{k}^{*}$, and expected fractions, *p*_*k*_, is quantified using the following "goodness" statistic:

$$\begin{array}{ccc} G=1-\frac{1}{K^{\prime }}\sum_{k=1}^{K^{\prime }}w_{k}\left|p_{k}^{*}-p_{k}\right| & \text{with} & 0\leq G\leq 1 \end{array}$$

where *w*_*k *_= 1 if $p_{k}^{*}$ > *p*_*k*_, and 2 otherwise. *K*' represents the discretization level of the computation. Twice more importance is given to deviations when $p_{k}^{*}$ <*p*_*k *_(inaccurate case). The weights penalize less the accurate case, which is the case where the fraction of true values falling into the *p*-probability interval is larger than expected. The goodness statistic is completed by the so-called "accuracy plot" that allows one to visualize departures between observed and expected fractions as a function of the probability *p*.

The accuracy plots in Figure 8C indicate that the geostatistical (both point and ATA Poisson kriging) and Bayesian approaches yield fairly similar results for realization #50. Although the BYM curve is the closest to the 45° line of perfect agreement, it also lies more frequently below that line (inaccurate case). Because this case is penalized more heavily in the computation of the goodness statistics, the three models end up with similar value for this criterion. Table 5 confirms the tendency for the BYM model to yield, on average over all 50 simulations, larger goodness statistic than the geostatistical model of uncertainty.

Not only should the true risk value fall into the PI according to the expected probability p, but this interval should be as narrow as possible to reduce the uncertainty about that value. In other words, among two probabilistic models with similar goodness statistic one would privilege the one with the smallest spread (more precise). Different measures of ccdf spread can be used: variance, interquartile range, and entropy. Following Goovaerts [43] the average width of the PIs that include the true value are plotted for a series of probabilities *p*. Once again, the geostatistical and Bayesian approaches yield fairly similar results for realization #50 (Figure 8D). Yet, the BYM-based probability intervals are slightly wider than the intervals based on the ATA kriging variance, a result that is consistent across most of the 50 simulations (Table 5). The larger precision of the geostatistical model of uncertainty agrees with the previous observation that the PK variance is smaller than the prediction variance calculated for the BYM model. Since the kriging variance decreases as the number of observations increases, the narrowest probability intervals are computed using Poisson kriging with 32 neighbors. The widest intervals are obtained using point Poisson kriging, which is expected since the point kriging variance is larger than the ATA kriging variance (recall Table 4).

The geostatistical model of uncertainty for realization #50 presents the advantage of yielding PIs that are slightly narrower than the Bayesian ones, while being more likely to include the true value (i.e. larger observed fractions). This property however is not captured by the separate computation of the goodness statistic and average PI width. A more informative criterion is the proportion of probabilities *p *for which the geostatistical PI is narrower than the Bayesian PI (PK width < BYM width) while including a larger fraction of true values (PK fraction > BYM fraction). This situation corresponds to the case where both blue and red ratio curves in Figures 8E–F exceed a value of one depicted by the horizontal dashed line. For realization #50, the point kriging model is jointly more accurate and precise than the BYM model 40% of the time, while the reverse is true only 7% of the time. The benefit of the geostatistical approach increases for ATA kriging: the smaller kriging variance results in narrower probability intervals hence increased precision (see red curve in Figure 8F). The ATA kriging model is jointly more accurate and precise than the BYM model 71% of the time, while the reverse is true only 1% of the time. The same conclusions hold on average across all 50 realizations. Table 5 shows that, according to this criterion, Poisson kriging outperform the Bayesian model across more than 80% of realizations, with the largest benefit displayed by ATA kriging for the heterogeneous Region 2.

#### Classification accuracy

By analogy with the classification conducted on observed cancer mortality rates in Figures 6E &6F, each simulated map underwent a classification into low and high-risk areas based on the probability of exceeding the area-wide rate. Figures 9A &9B present the results for the 50^th ^realization of Region 2 and a 0.75 probability threshold. Like in Figures 6E &6F, the geostatistical model leads to declaring substantially more counties as having elevated risk: 40 instead of 30 for the BYM model. The availability of true mortality risks here allows the computation of the number of false positives (declaring a county as having elevated risk when in fact its underlying true risk is smaller than the area-wide rate) and negatives (declaring a county to be in the background when in fact its underlying true risk exceeds the area-wide rate). The BYM model generates fewer false positives (2 instead of 6), which is expected since much less counties are flagged as having elevated risk. This result is however achieved at the expense of many more false negatives: 17 instead of 11 for ATA Poisson kriging. The geostatistical model thus leads to a smaller total number of misclassified counties. The same trend is observed when the risk threshold is up to 75% larger than the area-wide rate (RR = 1.75, Figures 9C &9D). Point and ATA Poisson kriging yield very similar proportions of errors.

**Figure 10:**
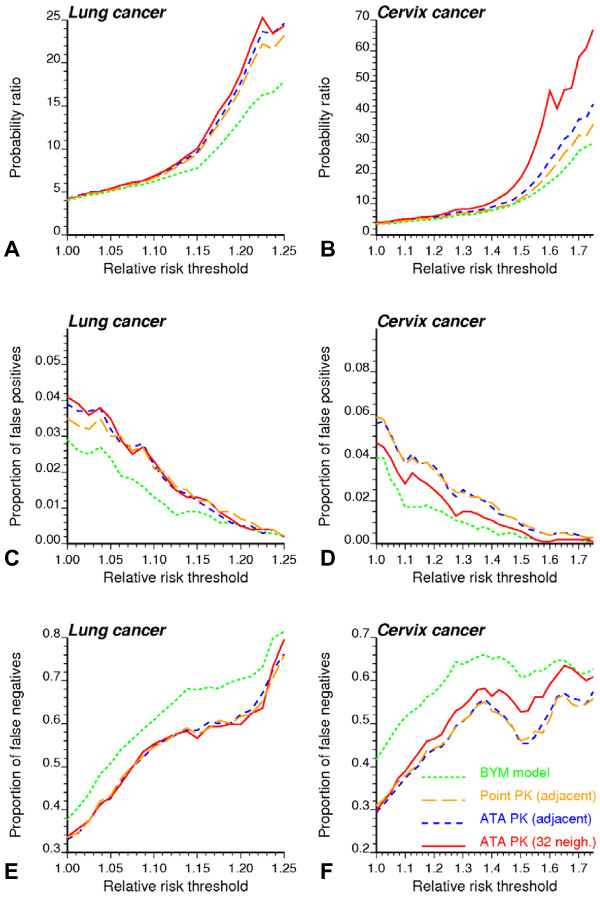
**Impact of modeling approach and risk threshold on the proportion of counties wrongly classified as having low or high cancer mortality risk**. Fifty realizations of the spatial distribution of cancer mortality rates were simulated and then analyzed using: Bayesian approach (BYM model), point Poisson kriging (PK) based on adjacent counties, and area-to-area Poisson kriging (ATA PK) based either on adjacent counties (same neighbors as BYM model) or the 32 closest counties in terms of distance between population-weighted centroids. The probability for each county to exceed a risk threshold proportional to the area-wide rate was averaged for counties that actually exceed or not that threshold. The ratio of these probabilities is a measure of discriminatory power and plotted as a function of the risk threshold (A, B). Counties with a probability larger than 0.75 are flagged as having significantly higher risk, and the resulting proportion of false positives (C, D) and negatives (E, F) are plotted as a function of the risk threshold.

Results averaged over all 50 realizations confirm that, regardless the type of cancer or risk threshold, the BYM model causes a much larger proportion of false negatives than the geostatistical model (Figures 10C–F). Although Poisson kriging generates more false positives, the probability of occurrence for this type of misclassification is one order of magnitude smaller than for the false negatives. To quantify the ability for different methods to discriminate high-risk from background areas, independently of the choice of a probability threshold, the probability of exceeding a risk threshold was averaged for two groups of counties: all counties with true risk above that threshold and all counties with true risk below that threshold. The larger the ratio of these two averaged probabilities, the higher the discriminatory power of the model. Figures 10A &10B indicate that probabilities computed using Poisson kriging allow the best discrimination of high-risk and background areas, in particular as the risk threshold is raised. Table 6 confirms the superiority of the geostatistical method for large risk thresholds: larger probability ratios are computed for 94 and 98% of lung and cervix cancer simulated maps, respectively. Point kriging is not as powerful as ATA kriging, in particular when the counties have very different shapes and sizes as in Region 2. Yet, the difference between the geostatistical and Bayesian approaches clearly cannot be explained simply by the lack of adjustment for the support effect in the BYM model.

Another way to compare the performances of Bayesian and geostatistical approaches, independently of the subjective choice of a probability threshold, is to compute the Receiver Operating Characteristic (ROC) curves. ROC curves plot the probability of false positive versus the probability of detection [44]. The probability of detection corresponds here to the proportion of true high-risk counties (i.e. counties with a mortality risk larger than a threshold) that are detected as the probability threshold decreases. In practice, the probability thresholds are identified with the probabilities of exceedence computed for these high-risk counties. For each of them the probability of false positive is calculated as the proportion of background counties that are wrongly declared as having elevated risks. Figure 9E &9F shows the ROC curve for the 50^th ^realization using a low (Relative Risk, RR = 1) and high risk threshold (RR = 1.25).

The most efficient approach is the one that allows the detection of a larger fraction of high-risk counties at the expense of fewer false positives; that is the ROC curve should be as close as possible to the vertical axis. For example, Figure 9E indicates that for both BYM and geostatistical models 60% of high-risk counties (i.e. counties with underlying true risk larger than the area-wide rate, RR = 1) are detected without causing any false positive. Differences between the two models increase when the risk threshold is raised to 125% the area-wide rate (RR = 1.25, Figure 9F). A quantitative measure of the detection error is the relative area above the ROC curve, which represents the average proportion of false positives (FP). According to this criterion, the geostatistical model outperforms the BYM model for higher risk thresholds: the average percentage of false positives is 5.10% for Poisson kriging and 7.81% for the BYM model. Similar results are observed across all 50 realizations, with a clear benefit of Poisson kriging for the least frequent cervix cancer. Once again, point Poisson kriging yields slightly more false positives than ATA kriging, in particular for Region 2.

## Conclusion

Since its early development for the assessment of mineral deposits, geostatistics has been used in a growing number of disciplines dealing with the analysis of data distributed in space and/or time. Its application to health data is fairly recent and, compared to the well established Bayesian modeling approach, the field of health geostatistics is still in its infancy. Nevertheless, the current geostatistical methodology offers a flexible framework to model the spatial variability of disease rates and estimate the underlying risk, taking into account not only the shape and size of geographical units, but also the spatial repartition of population within those units [15]. The approach described in this paper has been fully automated in TerraSeer's STIS software [29]: the "behind-the-scene" estimation, modeling, and deconvolution of risk semivariogram, as well as the automatic discretization of geographical units, makes Poisson kriging estimators as straightforward as empirical Bayesian smoothers while providing a measure of uncertainty in the form of the kriging variance. Furthermore, the comparison of point and area-to-area (ATA) Poisson kriging demonstrated the benefits of the geostatistical approach even under a simplified implementation whereby each geographical unit is assimilated with its population-weighted centroid.

Over the years, statisticians have developed models of increasing complexity, combining fixed effects with both uncorrelated and spatially structured random effects, leading to mixed effects or hierarchical models. The rich class of Bayes models yields the full posterior distribution of the risk while accounting for the uncertainty in the parameters of the model. Implementation of these sophisticated methods however is still cumbersome and relies on strong distributional assumptions (e.g. lognormal hypothesis), while parameter estimation requires time-consuming iterative procedures. Another drawback is that the full Bayesian modeling approach is overwhelmingly used with the conditional auto-regressive (CAR) model for defining the random effect associated with spatial autocorrelation. This computationally convenient choice is reasonable if all geographical entities are of similar size and arranged in a regular pattern but it is not particularly attractive otherwise [12]. In particular, it prohibits any change of support and creation of isopleth maps of risk, an operation easily conducted within the framework of area-to-point kriging [25].

The objectives of this paper were two-fold: 1) to illustrate the major differences between Poisson kriging and the Bayesian disease-mapping approach in two contrasted county geographies, and 2) to present the first simulation-based comparison of performance of Poisson kriging and full Bayesian models. The comparison study is certainly not exhaustive and only the BYM model, among the many alternative formulations available for disease mapping [2,14], was considered. Yet, studies have demonstrated the robustness of the BYM model which remains the favorite among practitioners [2,45].

The analysis of lung and cervix cancer mortality rates highlighted the stronger smoothing effect of the BYM model: geostatistical risk estimates tend to be lower in low-valued areas, while they exceed BYM predictions in high-valued areas. The use of BYM results to guide surveillance and control activities will thus lead to declaring substantially fewer counties as having elevated risk. These results confirm previous studies that showed Bayesian disease-mapping models to be conservative [14]. The impact of the modeling procedure was the largest for the prediction variances that differ in both magnitude and spatial pattern. As expected, the prediction variance increases as the population at risk, hence the reliability of raw rates, decreases. Because it accounts for the shape and size of counties, the Poisson kriging variance also increases for larger spatial supports, i.e. counties of large extent. On the other hand, the lognormal hypothesis causes the BYM prediction variance to increase with the estimated risk.

According to our simulation studies, the geostatistical approach yields smaller prediction errors, more precise and accurate probability intervals, and allows a better discrimination between counties with high and low mortality risks. The BYM model also generates smoother risk surfaces, leading to a much larger proportion of false negatives than the geostatistical model in particular as the risk threshold raises. The benefit of Poisson kriging is the largest for cervix cancer measured in Region 2 because of the combined effect of the low reliability of mortality rates and the wide range of county shapes and sizes. In general, the performance of Poisson kriging improves when data beyond the adjacent counties are used in the estimation.

The smoothing effect of Poisson kriging is controlled by the number of neighbors used in the estimation (more neighbors induce more smoothing), the population size (unreliable rates computed from small populations are smoothed more), and the variogram model (larger nugget effect induces more smoothing). On the other hand, the smoothing effect of the Bayesian model depends on the choice of a neighborhood weight structure (i.e. adjacency-based versus distance-based spatial weights [13]), the population size (unreliable rates computed from small populations are smoothed more), the type of distribution for the relative risk (mean-based smoothing using the Gaussian distribution versus median-based smoothing for double-exponential distribution), and the type of spatial model for the risk surface (i.e. continuous surface for the BYM model versus discontinuous surface for the partition and spatial mixture models). Both Bayesian and kriging estimators in this paper are mean-based smoothers and use the same adjacent neighbors. Estimating the nugget effect of a semivariogram is always somewhat subjective, in particular when only areal data are available which precludes any sample information on the short-range variability. The nugget effect of the point-support semivariogram model was here set to zero to facilitate the convergence of the deconvolution procedure [33]. Relaxing this constraint increases the smoothing effect of Poisson kriging that becomes comparable to the BYM model in Region 1 (results now shown). It has however no impact on the results for Region 2, where discrepancies between the dispersion variances of kriging and BYM model estimates were the largest. An alternative would be to adopt semi-parametric Bayesian spatial models that allow discontinuities in the risk surface and typically produce less smoothing than the BYM model [2]. Yet, these models are less robust [45] and are unlikely to benefit the simulation results since all realizations are based on continuous risk surfaces generated using semivariograms with no nugget effect.

The spatial support (i.e. point versus area) has a much smaller impact on the results than the statistical methodology (i.e. geostatistical versus Bayesian models). The trade-off cost for the easier implementation of point Poisson kriging is slightly larger kriging variances, which reduces the precision of the model of uncertainty and the ability to discriminate between background and risk-raised areas. The major drawback still remains the inability of point kriging to conduct any change of support and creation of isopleth maps of risk, an operation easily conducted within the framework of area-to-point kriging.

A critical element of any simulation-based comparison study is the assumptions used to generate the reference risk maps. In the present study, the mortality rates (ratio of number of deaths over population size) were not directly simulated using a geostatistical procedure, but rather were generated according to a Poisson distribution, which is similar to the approach used in other comparison studies of Bayesian models [2,14]. Because the BYM model is not designed to pick up discontinuities in the risk surface, all simulations were generated using smooth risk surfaces. The ability of Poisson kriging to uncover more complex risk structures should be investigated in future simulation studies which will include other classes of Bayesian hierarchical spatial models that generate less smoothing.

## Competing interests

The first author is affiliated with BioMedware a research company that also develops software for the exploratory spatial and temporal analysis of health and environmental data. With funding from the National Cancer Institute, the first author developed STIS (Space-Time Intelligence System), which is a commercial product of TerraSeer and will include a Poisson kriging function in a future release. Information about STIS software, as well as a beta-version of the geostatistical module, can be obtained by contacting the first author.

## Authors' contributions

PG carried out all simulation studies and drafted the manuscript. SG conducted the Bayesian analysis of real and simulated data using WinBUGS. All authors read and approved the final manuscript.
